# Impaired long-term memory retention and working memory in sdy mutant mice with a deletion in Dtnbp1, a susceptibility gene for schizophrenia

**DOI:** 10.1186/1756-6606-1-11

**Published:** 2008-10-22

**Authors:** Keizo Takao, Keiko Toyama, Kazuo Nakanishi, Satoko Hattori, Hironori Takamura, Masatoshi Takeda, Tsuyoshi Miyakawa, Ryota Hashimoto

**Affiliations:** 1 Division of Systems Medical Science, Institute for Comprehensive Medical Science, Fujita Health University, Toyoake, Aichi, Japan; 2 Genetic Engineering and Functional Genomics Unit, Frontier Technology Center, Kyoto University Graduate School of Medicine, Kyoto, Japan; 3 Japan Science and Technology Agency, CREST (Core Research for Evolutionary Science and Technology), Kawaguchi, Saitama, Japan; 4 Japan Science and Technology Agency, BIRD (Institute for Bioinformatics Research and Development), Kawaguchi, Saitama, Japan; 5 Department of Mental Disorder Research, National Institute of Neuroscience, National Center of Neurology and Psychiatry, Kodaira, Tokyo, Japan; 6 Department of Psychiatry, Osaka University Graduate School of Medicine, Suita, Osaka, Japan; 7 The Osaka-Hamamatsu Joint Research Center for Child Mental Development, Suita, Osaka, Japan

## Abstract

**Background:**

Schizophrenia is a complex genetic disorder caused by multiple genetic and environmental factors. The dystrobrevin-binding protein 1 (DTNBP1: dysbindin-1) gene is a major susceptibility gene for schizophrenia. Genetic variations in DTNBP1 are associated with cognitive functions, general cognitive ability and memory function, and clinical features of patients with schizophrenia including negative symptoms and cognitive decline. Since reduced expression of dysbindin-1 has been observed in postmortem brains of patients with schizophrenia, the sandy (sdy) mouse, which has a deletion in the Dtnbp1 gene and expresses no dysbindin-1 protein, could be an animal model of schizophrenia. To address this issue, we have carried out a comprehensive behavioral analysis of the sdy mouse in this study.

**Results:**

In a rotarod test, sdy mice did not exhibit motor learning whilst the wild type mice did. In a Barnes circular maze test both sdy mice and wild type mice learned to selectively locate the escape hole during the course of the training period and in the probe trial conducted 24 hours after last training. However, sdy mice did not locate the correct hole in the retention probe tests 7 days after the last training trial, whereas wild type mice did, indicating impaired long-term memory retention. A T-maze forced alternation task, a task of working memory, revealed no effect of training in sdy mice despite the obvious effect of training in wild type mice, suggesting a working memory deficit.

**Conclusion:**

Sdy mouse showed impaired long-term memory retention and working memory. Since genetic variation in DTNBP1 is associated with both schizophrenia and memory function, and memory function is compromised in patients with schizophrenia, the sdy mouse may represent a useful animal model to investigate the mechanisms of memory dysfunction in the disorder.

## Background

Schizophrenia is a complex genetic disorder characterized by profound disturbances of cognition, emotion and social functioning. DTNBP1 (dystrobrevin binding protein 1; dysbindin-1) has been one of the most studied and promising schizophrenia susceptibility genes [1-3]. Postmortem brain studies have demonstrated reduced expression of dysbindin-1 protein and mRNA in the schizophrenic brain [4-6]. DTNBP1 risk haplotypes for schizophrenia have been associated with decreased gene expression, whereas DTNBP1 protective haplotypes for the disorder have been associated with increased gene expression [7]. Furthermore, chronic treatment of mice with antipsychotics was not found to affect the expression levels of dysbindin-1 protein and mRNA in their brains [6,8], suggesting that prior evidence of lower dysbindin-1 protein and mRNA levels in the postmortem brains of schizophrenics is not likely to be an artifact of antemortem drug treatment. Together, these data indicate that the dysbindin-1 gene may confer susceptibility to schizophrenia through reduced expression.

Dysbindin-1 is expressed relatively ubiquitously in the brain, localized to neuronal cell bodies. It is expressed in regions implicated in schizophrenia, including the frontal cortex, temporal cortex, hippocampus, caudate, putamen, nucleus accumbens, amygdala, thalamus, and midbrain [5]. It may be involved in glutamatergic and dopaminergic function related to the pathophysiology of schizophrenia [9-13]. As the behavioral level, a genetic variation of DTNBP1 was reported to influence general cognitive ability and to be associated with cognitive decline in schizophrenia [14,15]. Memory function, one of the representative neurobiological traits related to the risk for developing schizophrenia, was also associated with genetic variations in DTNBP1 [16,17]. Moreover, the association between some clinical features of schizophrenia, such as its negative symptoms, and a risk haplotype of DTNBP1 has been demonstrated [18,19]. Risk genetic variations in DTNBP1, therefore, might be related to the cognitive functions affected in schizophrenia.

Obtaining an animal model of schizophrenia is extremely important in investigating the pathogenesis and treatment of the disease [20,21]. If a specific gene is suggested to be involved in schizophrenia by human genetic studies, the role of the gene should be examined in detail by using animals that carry abnormal expression and/or function of the genes [22]. Several mice with mutations in putative schizophrenia susceptibility genes have been shown to exhibit behavioral abnormalities reminiscent of schizophrenia [23-28]. Improved animal models of schizophrenia will provide valuable advances in the treatment of patients with the disorder.

Recently, we provided the first report of a behavioral analysis of the sandy (sdy) mutant mouse, which expresses no dysbindin-1 protein owing to a deletion in the dysbindin-1 gene [9]. Sdy was reported as a mutant mouse with diluted pigmentation that arose spontaneously in the DBA/2J inbred mouse strain and has simultaneous defects in melanosomes, lysosomes and platelet dense granules [29]. The sdy mice showed less activity and spent less time in the center of an open field apparatus [9]. Consistent with the latter observation, sdy mice also displayed evidence of heightened anxiety-like responses and deficits in social interaction [9]. However, cognitive ability has not been examined in sdy mice, although human genetic studies have consistently shown the effects of DTNBP1 genotypes on human cognitive function. Therefore, we performed a battery of behavioral analyses including memory performance in sdy mice.

## Results

### General behavioral characteristics of sdy mice

To address the behavioral effects of Dtnbp1 deficiency, we subjected sdy mutant mice to a comprehensive behavioral test battery that covers many distinct behavioral domains, from simple sensorimotor functions to higher brain functions, including learning and memory. We present here results showing significant impact of Dtnbp1 deficiency. The raw data of behavioral tests, which are not described in this paper, are disclosed in the gene-brain-phenotyping database . The results of social interaction, hot plate test, acoustic startle response and its prepulse inhibition and the passive avoidance test are open to the public in the database. Sdy mice did not differ significantly from wild type mice in overall health and appearance, body weight (wild type, 25.09 ± 0.386 g; sdy, 24.985 ± 0.623 g, F(1, 38) = 0.021, p = 0.8868; genotype effect), or core body temperature (wild type, 36.8 ± 0.146°C; sdy, 36.445 ± 0.121°C, F(1, 38) = 3.509, p = 0.0688; genotype effect). In addition, there was no significant difference between sdy mice and wild type mice in sensory-motor reflex (eye blink, ear touch, whisker twitch, righting reflex; data not shown) or muscular strength assessed in grip strength test (wild type, 0.623 ± 0.023 N; sdy, 0.675 ± 0.02 N, F(1, 38) = 3.037, p = 0.0895) and wire hang test (wild type, 42.05 ± 3.774 sec; sdy, 38.65 ± 4.234 sec, F(1, 38) = 0.359, p = 0.5524).

### Locomotor activity and motor coordination of sdy mice

To examine spontaneous locomotor activity and response to a novel environment, sdy mice and wild type mice were assayed in an open field test. Sdy mice showed decreased locomotor activity and exploratory behavior (distance traveled in 120 min: wild type, 5829.850 ± 665.814 cm; sdy, 4208.250 ± 432.967 cm, F(1, 38) = 4.220, p = 0.0469) (Additional figure 1A). There was no significant difference in the vertical activity, stereotypic behavior or time spent in the center area in the open field test (Additional figure 1B, C, and 1D).

Decreased locomotor activity and exploratory behavior were also detected in the light/dark transition test (Additional figure 2A, B). There was a significant genotype difference in distance traveled in the dark box (F(1, 38) = 21.437, p < 0.0001) and time course for the decrease in distance traveled in the dark box was significantly different between genotypes (F(9, 342) = 1.958, p = 0.0434) (Additional figure 2B). There was no significant difference in time spent in the light box, often used as an index of anxiety-like behavior. We also conducted an elevated plus maze test to assess anxiety-like behaviors and no significant difference between genotypes was observed (Additional figure 3).

**Figure 1:**
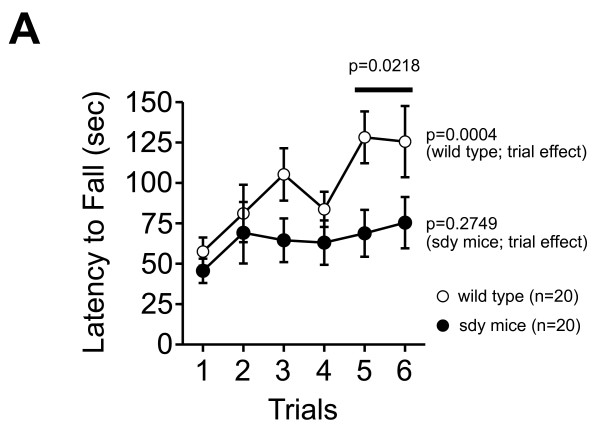
**Motor coordination deficit of sdy mice**. Latency to fall (second) from the rotating drum was counted in wild type mice and sdy mice in a rotarod test. First 3 trials were conducted on the first day, and later 3 trials were conducted on the second day. The trial effects of each genotype were analyzed by one-way repeated measures ANOVA and the genotype effect in 5th and 6th trial was analyzed by two-way repeated measures ANOVA.

In a rotarod test, wild type mice demonstrated significant improvement in latencies to fall (F(5, 95) = 5.024, p = 0.0004; trial effect), which was not evident in the sdy mice (F(5, 95) = 1.290, p = 0.2749; trial effect) (Figure 1). Since the effect of motor learning reached a plateau in wild type mice after the 5th trial, we compared the performance of each genotype in the 5th and 6th trials. In these trials, there was a significant difference in latency to fall between sdy and wild type mice (F(1,38) = 5.720, p = 0.0218; genotype effect). In addition, sdy mice showed a swimming deficit in a Porsolt forced swim test, where more sdy mice drawn to death than wild type mice (wild type, 0 out of 8 mouse was died; sdy, 3 out of 8 mice were died, Fisher's exact test, p = 0.20). While this difference did not reach statistical significance, an effect may have been seen if we had stopped the experiment prematurely due to the drastic consequences. We suspect the reason for the sdy mice drowning in the experiment may have been due to deficits in swimming ability and/or exercise performance.

### Performance in the Barnes circular maze test

Long term spatial memory, which is dependent on the functioning of the hippocampus, was assessed in sdy mice and wild type mice using a Barnes circular maze [30-32]. The task is similar to the Morris water maze as both tests require an escape response. The Barnes maze test was chosen for this study since it does not involve swimming like the Morris water maze [30-32]. Given the possible motor deficits in sdy mice, swimming ability might have given an advantage to wild type mice over sdy mice in the Morris water maze.

**Figure 2:**
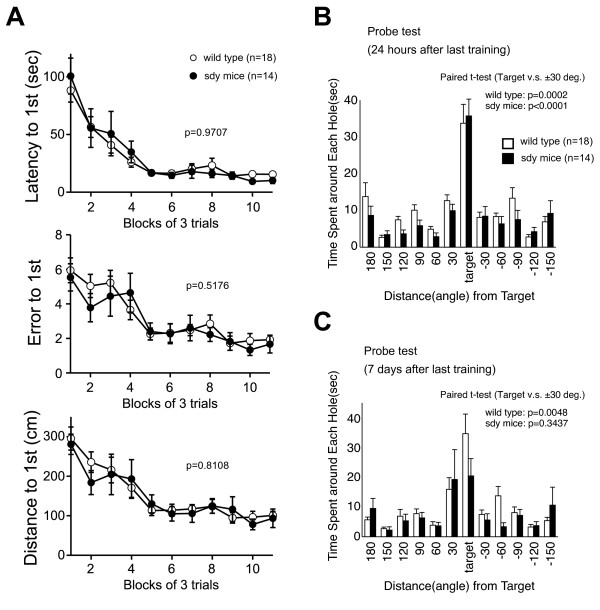
**Deficit of long-term memory retention in sdy mice**. (A) Latency to reach the target hole (up), numbers of errors (middle) and distance to reach the target hole (bottom) across training were recorded. Data were analyzed by two-way repeated measures ANOVA. Data are presented as averages of 3 trials. (B) Time spent around each hole in the probe trial conducted 24 hours after last training. (C) Time spent around each hole in the probe trial conducted 7 days after last training. Time spent around target hole and holes adjacent to the target were compared by paired t-test.

Both sdy mice and wild type mice learned to locate the escape hole during the course of the training period as indicated by a progressive reduction in latencies and numbers of errors to escape (wild type, F(32, 512) = 2.896, p < 0.0001, sdy, F(32, 448) = 2.806, p < 0.0001; trial effect was analyzed by one-way repeated measures analysis of variance (ANOVA)). Through the training trials, there were no statistical differences between sdy mice and wild type mice in latencies (F(1, 30) = 0.001, p = 0.9707; genotype effect), errors (F(1, 30) = 0.429, p = 0.5176; genotype effect), and distances (F(1, 30) = 0.058, p = 0.8108; genotype effect) to escape through the target hole (Figure 2A).

The probe trial was conducted 24 hours after the last training session. Both sdy mice and wild type mice selectively located the correct target hole where the escape box had been and both sdy mice and wild type mice spent significantly more time around the target hole compared to the holes adjacent to the target (paired t-test, wild type: t(17) = 4.645, p = 0.0002; sdy mice: t(13) = 6.538, p < 0.0001) (Figure 2B). To assess the long-term retention of spatial memory in sdy mice, we also conducted probe tests 7 days after the last training trial. During the retention probe test, wild type mice selectively located the correct target hole where the escape box had been and spent significantly more time around the target hole compared to the adjacent holes (paired-test, t(17) = 3.239, p = 0.0048), but sdy mice did not (paired-test, t(13) = 0.983, p = 0.3437)(Figure 2C). These results indicate that sdy mice are impaired in memory retention rather than memory recall.

### Performance in the T-maze forced alternation task

**Figure 3:**
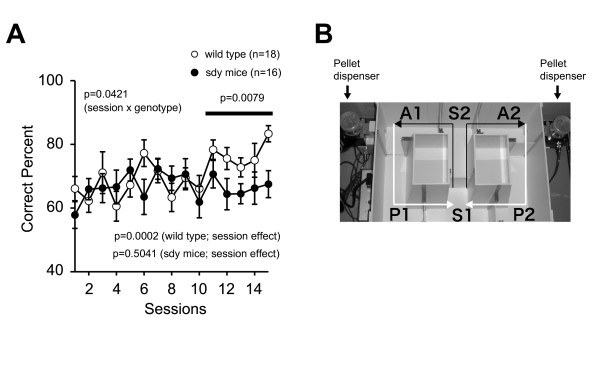
**Working memory deficit in sdy mice**. The percent of correct choices in T-maze forced alternation task was presented. Data were analyzed by two-way repeated measures ANOVA. Improvements of performance over training were analyzed one-way repeated measures ANOVA. (B) Apparatus of the T-maze forced alternation task.

We next examined a T-maze forced alternation task in sdy mice and wild type mice, a task of working memory [33-35]. Wild type mice improved their performance over training as measured by an increase in the number of correct choices made (F(14, 238) = 3.067, p = 0.0002; session effect), while sdy mice did not (F(14, 210) = 0.952, p = 0.5041; session effect). There was a significant session by genotype interaction effect on the percent of correct choices (F(14, 448) = 1.760, p = 0.0421). After acquisition trials, sdy mice demonstrated significantly less correct choices made than wild type mice (F(1, 32) = 8.031, p = 0.0079; from 11^th ^to 15^th ^session) (Figure 3A).

To increase the difficulty of the task, a delay period (10, 30, 60 sec) was applied. Under these conditions, there was no significant difference in the percentage of correct choices made between sdy and wild type mice (10 sec delay, F(1, 32) = 0.041, p = 0.8412; 30 sec delay, F(1, 32) = 1.096, p = 0.3030; 60 sec delay, F(1, 32) = 0.479, p = 0.4939). However, DBA/2J, the background strain of sdy mice, has been reported to demonstrate relatively lower performance of working memory in radial maze compared to other inbred strains such as C57BL/6J and BALB/cJ [36]. Therefore, increasing the difficulty of the task with a delay period may have masked the effect of genotype.

## Discussion

The lack of *Dtnbp1 *did not lead to obvious abnormalities in overall health, appearance, sensory-motor reflex or muscular strength. However, sdy mice demonstrated abnormal behaviors some of which are reminiscent of schizophrenia. Firstly, sdy mice showed hypolocomotor activity, which is consistent with our previous report on the behavior of sdy mice [9]. In most animal models of schizophrenia, hyperlocomotor activity is considered of potential relevance to the positive symptoms of schizophrenia. Speculatively, the decreased activity, shown by sdy mice, could be related to negative symptoms of schizophrenia.

In addition, we found poor motor learning in the rotarod test. It is known that motor skill learning assessed by rotary pursuit is impaired in patients with schizophrenia [37,38]. One possibility is that deficit in motor learning could be part of a pattern of generalized neuropsychological impairment in schizophrenic patients. Such an explanation could also apply to the sdy mice since they demonstrated cognitive deficits in the present study as well as learning deficits. Another possible explanation for the motor learning deficits in sdy mice is that they reflect poor motor coordination, likely to have affected the sdy mice since they also exhibited swimming deficits in the Porsolt forced swim test. Neuromotor dysfunction is consistently found in schizophrenia [39-41]. Dysbindin-1, which binds to dystrobrevin, is expressed in neuromuscular junctions and cerebellar tissues [42]. Similar to our results of motor coordination deficits in sdy mice, dystrobrevin knockout mice also show poor motor coordination [42].

In addition to abnormal motor related behaviors, sdy mice also displayed cognitive impairments including impaired memory retention and working memory. Memory function is one of the representative neurobiological traits of schizophrenia and is deeply disturbed in patients with the disorder [43,44]. Although previous study reported enhanced anxiety-like behavior in sdy mice [9], sdy mice did not show altered anxiety-like behavior in the present study. For example, in the previous study, apparatus with a black walk way was used in the elevated plus maze test, while we used apparatus with white walk way. The illumination level and time behavior was observed for was also different between studies. Hattori et al. examined behavior for 20 minutes, while we examined for only 10 minutes. Consistent with our results, however, anxiety-like behavior was unaltered in sdy mice within the first 10 minutes of the Hattori et al study, suggesting we may have observed anxiety-related changes had we observed the mice for longer. On the other hand, sdy mice showed decreased locomotor activity in open field test (Additional figure 1) and light/dark transition test (Additional figure 2). In light/dark transition test, the time course of distance traveled in the dark box was different between genotypes (Additional figure 2B), suggesting abnormal habituation to a novel environment in sdy mice. This reduction of locomotor activity in sdy mice caused by novel and stressful environment may affect anxiety-like behavior in the previous study.

Overall therefore, the data presented show deficits in exploratory activity and memory which could be consistent with the negative and cognitive symptoms of schizophrenia and with neuropsychological and neuromotor deficits seen in the disorder. However, the schizophrenia phenotype is unlikely to be simulated in animal models through the disruption of a single gene, and it should be pointed out that similar behavioral effects can be seen following the disruption of genes thought to have little to do with schizophrenia. Notwithstanding, the phenotypes in the sdy mice may be useful for understanding the neurobiological role of dysbindin and may be relevant to particular endophenotypes of schizophrenia.

### Cognitive deficits of sdy mice as an animal model of cognitive dysfunctions of schizophrenia

As dysbindin-1 is a susceptibility gene for schizophrenia, sdy mice could be a good animal model of the disorder. In the present study, we demonstrated that sdy mice displayed cognitive deficits including impaired long-term memory retention in the Barnes maze test and impaired working memory in the T-maze forced alternation task. Cognitive dysfunctions, including impaired attention, decreased working memory and decreased long term memory retention, are described as core symptoms of schizophrenia [45]. Cognitive deficits have also commonly been observed in other pharmacological, neurodevelopmental, and genetic animal models for schizophrenia [46,47]. Recently, mice with mutations in several schizophrenia susceptibility genes have been shown to have cognitive deficits. For example, mice with mutations in Neuregulin-1, Disrupted in schizophrenia 1 (DISC1) and calcineurin, all identified as risk genes for schizophrenia [3,48,49], show impaired working memory. [25,50-52]. YWHAE, a binding partner of DISC1, was also identified as a susceptibility gene for schizophrenia and heterozygous knockout mice of this gene also causes working memory deficits [24]. Mutant mice relevant to both the dopamine and glutamate hypotheses of schizophrenia similarly show working memory deficits, including mice lacking D2 and D3 receptors [53], mice with transient striatal overexpression of D2 [54], GluR1 knockout mice [55] and mice with dentate gyrus specific NMDA receptor knockout [56]. In addition, we have demonstrated working memory deficits in knockout mice for the alpha-isoform of calcium/calmodulin dependent kinase (alpha-CaMKII) [28], a major signaling molecule downstream of NMDARs associated with both schizophrenia and working memory in humans [57]. Thus several diverse animal models show behavioral abnormalities in memory function in common with the sdy mouse. This suggests that the sdy mouse may have not only construct validity but also reasonable face validity, as a model of the cognitive dysfunctions of schizophrenia.

### Why does Dysbindin-1 deficiency cause impairments in working memory and long term memory retention?

Several lines of evidence indicate that the dentate gyrus and mossy fiber terminus play important roles in working memory and long term memory retention [58-61]. Dentate gyrus is activated by spatial working memory tasks [62,63] and others have reported a highly positive correlation between spatial working memory performance and the size of the mossy fiber terminals [64,65]. Likewise, Ramirez-Amaya and colleagues show mossy fiber synaptogenesis correlates with performance of spatial long-term memory retention in a water maze test [66]. We recently reported that continuous neurogenesis in dentate gyrus is essential for long-term memory retention [67]. In addition, mossy fiber – CA3 synapses exhibit long-term plasticity phenomena, such as long term potentiation [68], which may contribute to hippocampal memory processing.

In reference to deficits of the dentate gyrus and spatial working memory, mutant mice of the DISC1 gene and the alpha-CaMKII gene should be noted. Mice lacking a C-terminal portion of DISC1 show morphological abnormalities in the dentate gyrus and deficits of spatial working memory [69]. Heterozygous knockout mice of alpha-CaMKII, which displayed a severe working memory deficit, had remarkable abnormalities in their dentate gyrus that is referred as immature dentate gyrus [28]. In the dentate gyrus of alpha-CaMKII heteroknockout mice, increased transmission and reduced frequency facilitation at the synapses between mossy fibers and CA3 pyramidal cells [28]. These mice with deficits in working memory and long term memory retention showed abnormalities in dentate gyrus. Abnormality in dentate gyrus of sdy mouse has not been reported, however, dysbindin-1 could play a critical role in memory disturbance in schizophrenia via dentate gyrus. Indeed, dysbindin-1 is expressed at highly levels in the dentate gyrus and mossy fibers [6,70]. Moreover, dysbindin-1 mRNA in the hippocampal formation of patients with schizophrenia shows reduced expression in dentate granule and polymorph cells and in CA3, but not in CA1 [4]. Talbot and colleagues similarly reported that the reduction of dysbindin-1 was relatively restricted in dentate gyrus and mossy fiber terminus of patients with schizophrenia [6]. This presynaptic reduction of dysbindin-1 protein was inversely correlated with increased expression of vesicular glutamate transporter-1, indicating glutamatergic alterations within intrinsic hippocampal formation connections in schizophrenia. We previously reported that dysbindin-1 plays a role in the glutamate neurotransmission [13]. Overexpression of dysbindin-1 induced the expression of two pre-synaptic proteins, SNAP25 and synapsin I, and increased extracellular basal glutamate levels and release of glutamate evoked by high potassium in primary cortical neuronal culture. Conversely, knockdown of endogenous dysbindin-1 protein by small interfering RNA (siRNA) resulted in the reduction of pre-synaptic protein expression and glutamate release, suggesting that dysbindin-1 might influence exocytotic glutamate release via upregulation of the molecules in pre-synaptic machinery. Consistent with this role of dysbindin-1, altered regulation of exocytosis and vesicle biogenesis in neurons has been reported in sdy mice [71]. This included specific defects in neurosecretion and vesicular morphology in hippocampal synapses such as larger vesicle size, slower quantal vesicle release, lower release probability, and smaller total population of the readily releasable vesicle pool. Collectively therefore, these data suggest that deficiency of dysbindin-1 could be linked to glutamatergic dysfunction in the dentate gyrus and mossy fibers, and this could possibly underpin cognitive deficits related to the dentate gyrus in both schizophrenia and sdy mice.

In addition to being implicated in glutamatergic neurotransmission, dysbindin is also highly expressed in dopaminergic nuclei [5,12]. A recent study found that *DTNBP1 *siRNA transfection reduced dysbindin-1 protein, increased surface expression of dopamine D2 receptor and blocked dopamine-induced internalization of dopamine D2 receptor in SH-SY5Y cells [11]. Dysbindin-1, via its role in BLOC-1, may thus regulate recycling of dopamine D2 receptor in postsynaptic targets of dopaminergic synapses [11]. Another study reported that dopamine release was increased by siRNA-mediated silencing of dysbindin-1 in PC12 cells [12]. We found that sdy mice displayed lower levels of dopamine in the cerebral cortex, hippocampus, and hypothalamus compared to wild type mice [9,10], further demonstrating that dysbindin-1 plays a crucial role in the dopaminergic system. Altered dopaminergic transmission in sdy mice could be related to the deficits of working memory, since mice deficient for dopamine D2 and D3 receptors and mice selectively overexpressing striatal dopamine D2 receptors show working memory deficits [53,54]. The former mice exhibited abnormal dopamine D1 receptor activity in the frontal cortex, and the latter mice also displayed altered dopamine levels, rates of dopamine turnover, and activation of D1 receptors in the frontal cortex. In addition, D1 receptor blockade in hippocampus – prefrontal cortex circuits has been shown to disrupt working memory in the rat [72,73]. These data therefore suggest that the memory impairment observed in sdy mice might be related to the dopaminergic system and dopaminergic projections to the frontal cortex. Indeed, dopamine has a major role in regulating the excitability of the cortical neurons upon which the working memory function of the prefrontal cortex depends [74].

## Conclusion

We have provided the first report of impaired long-term memory retention and working memory in sdy mutant mice, which lack the dysbindin-1 gene, a susceptibility gene for schizophrenia. The behavioral phenotype shows similarities with several other genetic animal models with mutations in putative schizophrenia susceptibility genes. Further studies to explore any shared mechanisms underpinning the cognitive deficits in the sdy mice and other genetic animal models of schizophrenia might provide novel insight into the pathophysiology of schizophrenia and new drug targets for the disorder.

## Methods

### Animals and experimental design

Sdy mice (dysbindin-1 mutant mice) were obtained from the Jackson Laboratory (Bar Harbor, Maine, USA). Mice were housed one per cage in a room with a 12-hr light/dark cycle (lights on at 7:00 a.m.) with access to food and water ad libitum. Behavioral testing was performed between 9:00 a.m. and 6:00 p.m. After the tests, the apparatus were cleaned with super hypochlorous water to prevent a bias due to olfactory cues. All behavioral tests were conducted in a manner similar to those described previously [75,76]. All behavioral testing procedures were approved by the Animal Care and Use Committee of Kyoto University Graduate School of Medicine.

### Neurological screen

Neurological screen was performed with 10-wk-old male mice. The righting, whisker touch, and ear twitch reflexes were evaluated. A number of physical features, including the presence of whiskers or bald hair patches, were also recorded.

### Neuromuscular strength

Neuromuscular strength was performed with 10-wk-old male mice, and tested with the grip strength test and wire hang test. A grip strength meter (O'Hara & Co., Tokyo, Japan) was used to assess forelimb grip strength. Mice were lifted and held by their tail so that their forepaws could grasp a wire grid. The mice were then gently pulled backward by the tail with their posture parallel to the surface of the table until they released the grid. The peak foce applied by the forelimbs of the mouse was recorded in Newtons (N). Each mouse was tested three times, and the greatest value measured was used for statistical analysis. In the wire hang test, the mouse was placed on a wire mesh that was then inverted and waved gently, so that the mouse gripped the wire. Latency to fall (second: sec) was recorded, with a 60 s cut-off time.

### Open field test

Locomotor activity was measured using an open field test. Open field test was performed with 11-wk-old male mice. Each mouse was placed in the center of the open field apparatus (40 × 40 × 30 cm; Accuscan Instruments, Columbus, OH). Total distance traveled (in cm), vertical activity (rearing measured by counting the number of photobeam interruptions), time spent in the center, the beam-break counts for stereotyped behaviors, and number of fecal boli were recorded. Data were collected for 120 min.

### Light/dark transition test

Light/dark transition test was performed as previously described [77]. The apparatus used for the light/dark transition test consisted of a cage (21 × 42 × 25 cm) divided into two sections of equal size by a partition containing a door (O'Hara & Co., Tokyo, Japan). One chamber was brightly illuminated (390 lux), whereas the other chamber was dark (2 lux). Mice were placed into the dark side and allowed to move freely between the two chambers with the door open for 10 min. The total number of transitions between chambers, time spent in each side, first latency to enter the light side and distance travelled were recorded automatically using Image LD software (see 'Image analysis').

### Elevated plus maze test

Elevated plus-maze test was performed as previously described [28]. The elevated plus-maze (O'Hara & Co., Tokyo, Japan) consisted of two open arms (25 × 5 cm) and two enclosed arms of the same size, with 15-cm high transparent walls. The arms and central square were made of white plastic plates and were elevated to a height of 55 cm above the floor. To minimize the likelihood of animals falling from the apparatus, 3-mm high plastic ledges were provided for the open arms. Arms of the same type were arranged at opposite sides to each other. Each mouse was placed in the central square of the maze (5 × 5 cm), facing one of the closed arms. Mouse behaviour was recorded during a 10-min test period. The numbers of entries into, and the time spent in open and enclosed arms, were recorded. The illumination level was 100 lux at the center of the maze. For data analysis, we used the following four measures: the percentage of entries into the open arms, the time spent in the open arms (s), the number of total entries, and total distance travelled (cm). Data acquisition and analysis were performed automatically using Image EP software (see 'Image analysis').

### Rotarod test

Motor coordination and balance were tested with a rotarod test. The rotarod test, using an accelerating rotarod (UGO Basile Accelerating Rotarod, Varese, Italy), was performed by placing 13-wk-old mice on rotating drums (3 cm diameter) and measuring the time each animal was able to maintain its balance on the rod. The speed of the rotarod accelerated from 4 to 40 rpm over a 5-min period.

### Porsolt forced swim test

The apparatus for Porsolt forced swim test consisted of four plastic cylinders (20 cm height × 10 cm diameter). The cylinders were filled with water (23°C) up to a height of 7.5 cm. Mice were placed into the cylinders, and their behavior were recorded over a 10-min test period.

### Barnes circular maze test

The Barnes task was conducted on "dry land," a white circular surface, 1.0 m in diameter, with 12 holes equally spaced around the perimeter (O' Hara & Co., Tokyo, Japan). The circular open field was elevated 75 cm from the floor. A black Plexiglas escape box (17 × 13 × 7 cm), which had paper cage bedding on its bottom, was located under one of the holes. The hole above the escape box represented the target, analogous to the hidden platform in the Morris task. The location of the target was consistent for a given mouse, but was randomized across mice. The maze was rotated daily, with the spatial location of the target unchanged with respect to the visual room cues, to prevent a bias based on olfactory or proximal cues within the maze. The first training was started when wild type mice and sdy mice were 34 weeks old. Three trials per day were conducted for 9 successive days in the beginning (on days 5 and 6, no trial was undertaken). One day after the last training, a probe trial was conducted without the escape box, to confirm that this spatial task was acquired based on navigation using distal environment room cues. Time of latency to reach the target hole, number of errors, distance to reach the target hole, and time spent around each hole were recorded by video tracking software (Image BM, see 'Image analysis').

### T-maze forced alternation task

The forced alternation task was conducted using an automatic T-maze that we devised (Figure. 3B, O'Hara & Co., Tokyo, Japan). It was constructed of white plastics runways with walls 25-cm high. The maze was partitioned off into 6 areas by sliding doors that can be opened downward. The stem of T was composed of area S2 (13 × 24 cm) and the arms of T were composed of area A1 and A2 (11.5 × 20.5 cm). Area P1 and P2 were the connecting passage way from the arm (area A1 or A2) to the start compartment (area S1). The end of each arm was equipped with a pellet dispenser that could provide food reward. The pellet sensors were able to record automatically pellet intake by the mice. One week before the pre-training, mice were deprived of food until their body weight was reduced to 80–85% of the initial level. Mice were kept on a maintenance diet throughout the course of all the T-maze experiments. Before the first trial, mice were subjected to three 10-min adaptation sessions, during which they were allowed to freely explore the T-maze with all doors open and both arms baited with food. On the day after the adaptation session, mice were subjected to a forced alternation protocol for 16 days (one session consisting of 10 trials per day; cutoff time, 50 min). The first training was started when wild type mice and sdy mice were 29 weeks old. Mice were given 10 pairs of training trials per day. On the first (sample) trial of each pair, the mouse was forced to choose one of the arms of the T (area A1 or A2), and received the reward at the end of the arm. Choosing the incorrect arm resulted in no reward and confinement to the arm for 10 sec. After the mouse consumed the pellet or the mouse stayed more than 10 sec without consuming the pellet, door that separated the arm (area A1 or A2) and connecting passage way (area P1 or P2) would be opened and the mouse could return to the starting compartment (area S1), via connecting passage way, by itself. In this way, the potential stress could be reduced compared to the traditional forced alternation paradigm in which human experimenter brings back the mouse to the start box by hand. The mouse was then given 3 sec delay there and a free choice between both T arms and rewarded for choosing the other arm that was not chosen on the first trial of the pair. The location of the sample arm (left or right) was varied pseudo-randomly across trials using Gellermann schedule so that mice received equal numbers of left and right presentations. A variety of fixed extra-maze clues surrounded the apparatus. On the 16–21th day, delay (10, 30 or 60 sec) was applied after the sample trial. Data acquisition, control of sliding doors, and data analysis were performed by Image TM software (see 'Image analysis').

### Image analysis

The applications used for the behavioral studies (Image LD, Image EP, Image BM, and Image TM) were based on the public domain NIH Image program (developed at the U.S. National Institutes of Health and available on the Internet at ) and ImageJ program , which were modified for each test by Miyakawa (available through O'Hara & Co., Tokyo, Japan).

### Statistical analysis

Statistical analysis was conducted using Stat View (SAS institute). Data were analyzed by two-way ANOVA, or two-way repeated measures ANOVA, unless noted otherwise. In Porsolt forced swim test, mortality of each genotype was analyzed by Fisher's exact test. Values in text and graphs were expressed as mean ± SEM. All p-values reported are two tailed. Statistical significance was defined as p < 0.05.

## List of abbreviations

alpha-CaMKII: Alpha-isoform of calcium/calmodulin dependent kinase; ANOVA: analysis of variance; DISC1: Disrupted-In-Schizophrenia 1; dysbindin-1 (DTNBP1): dystrobrevin binding protein 1; siRNA: small interfering RNA.

## Competing interests

The authors declare that they have no competing interests.

## Authors' contributions

KTa carried out the behavioral studies of mice, performed the statistical analysis and wrote the manuscript. KTo, KN, SH and HT carried out the behavioral studies of mice. MT and TM participated in the design and coordination of the study and helped to draft the manuscript. RH supervised the entire project, wrote the manuscript, was critically involved in the design, analysis and interpretation of the data and was responsible for performing the literature review. All authors read and approved the final manuscript.

## Supplementary Material

Additional file 1 **Reduced locomotor activity in sdy mice in an open field test.** (A) Total locomotor distance. (B) Count of vertical activity. (C) Time spent on the centre of the field. (D) Count of stereotypic behavior. Data were analyzed by two-way repeated measures ANOVA. Click here for file

Additional file 2 **Reduced locomotor activity in sdy mice in a light/dark transition test.** (A) Distance traveled in the light and dark boxes. (B) Time course of the distance traveled in the dark box. (C) Time spent in the light box. (D) Number of transitions between the light and dark boxes. (E) Latency of first entry into light box. Data were analyzed by two-way ANOVA and two-way repeated measures ANOVA. Click here for file

Additional file 3 **Normal anxiety-like behavior in sdy mice in elevated plus maze test.** (A) Total number of arm entries. (B) Percentage entries into open arms. (C) Distance traveled. (D) Percentage entries into open. Data were analyzed by two-way ANOVA and two-way repeated measures ANOVA. Click here for file
