# Supplementary figures and images for: Impaired long-term memory retention and working memory in sdy mutant mice with a deletion in Dtnbp1, a susceptibility gene for schizophrenia
Source: Mol Brain. 2008 Oct 22;1:11. doi: 10.1186/1756-6606-1-11 (PMC2584096; doi:10.1186/1756-6606-1-11)

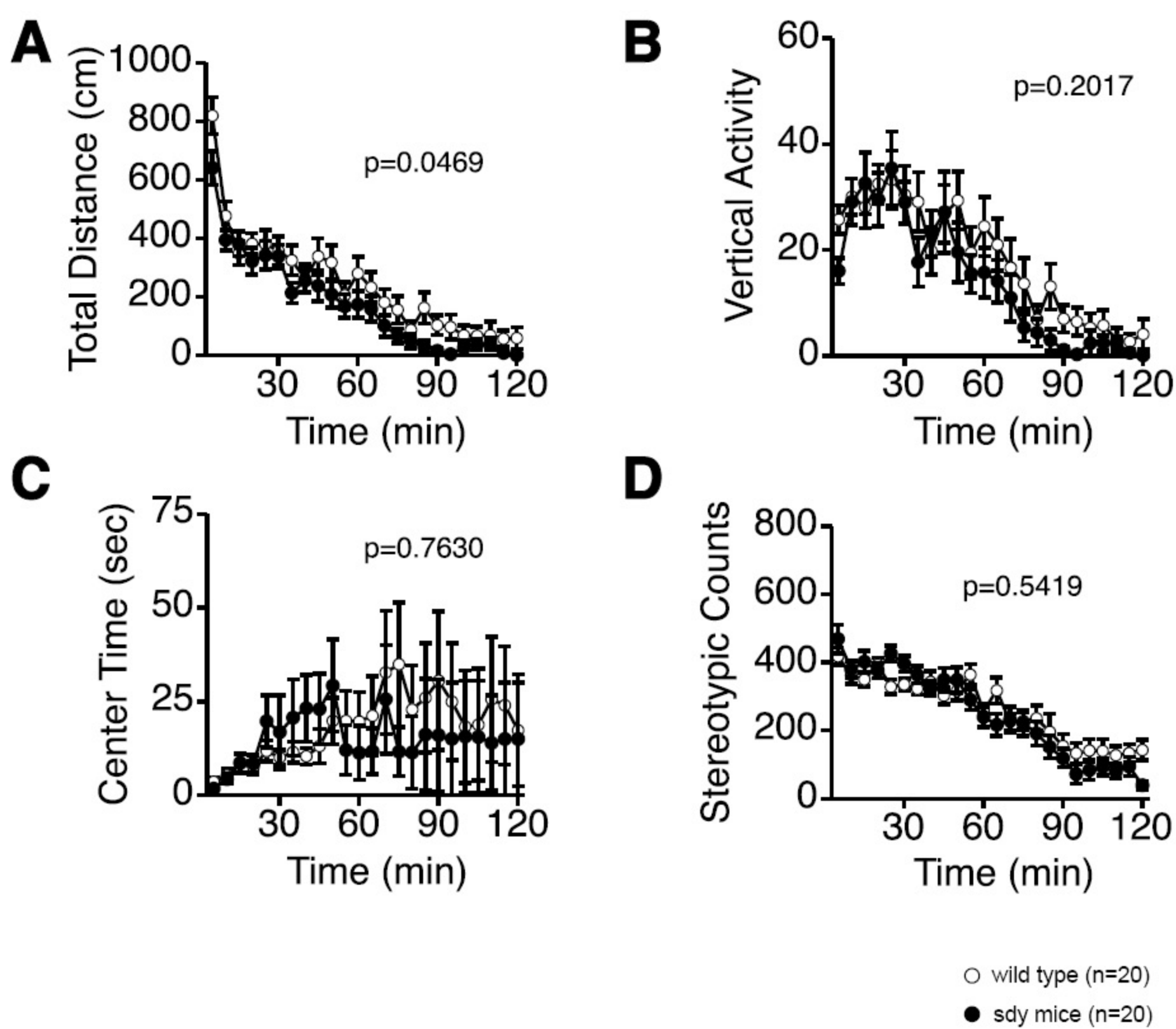

Additional Figure 1

Supplement: Additional file 1 — Reduced locomotor activity in sdy mice in an open field test. (A) Total locomotor distance. (B) Count of vertical activity. (C) Time spent on the centre of the field. (D) Count of stereotypic behavior. Data were analyzed by two-way repeated measures ANOVA. [file 1756-6606-1-11-S1.pdf]

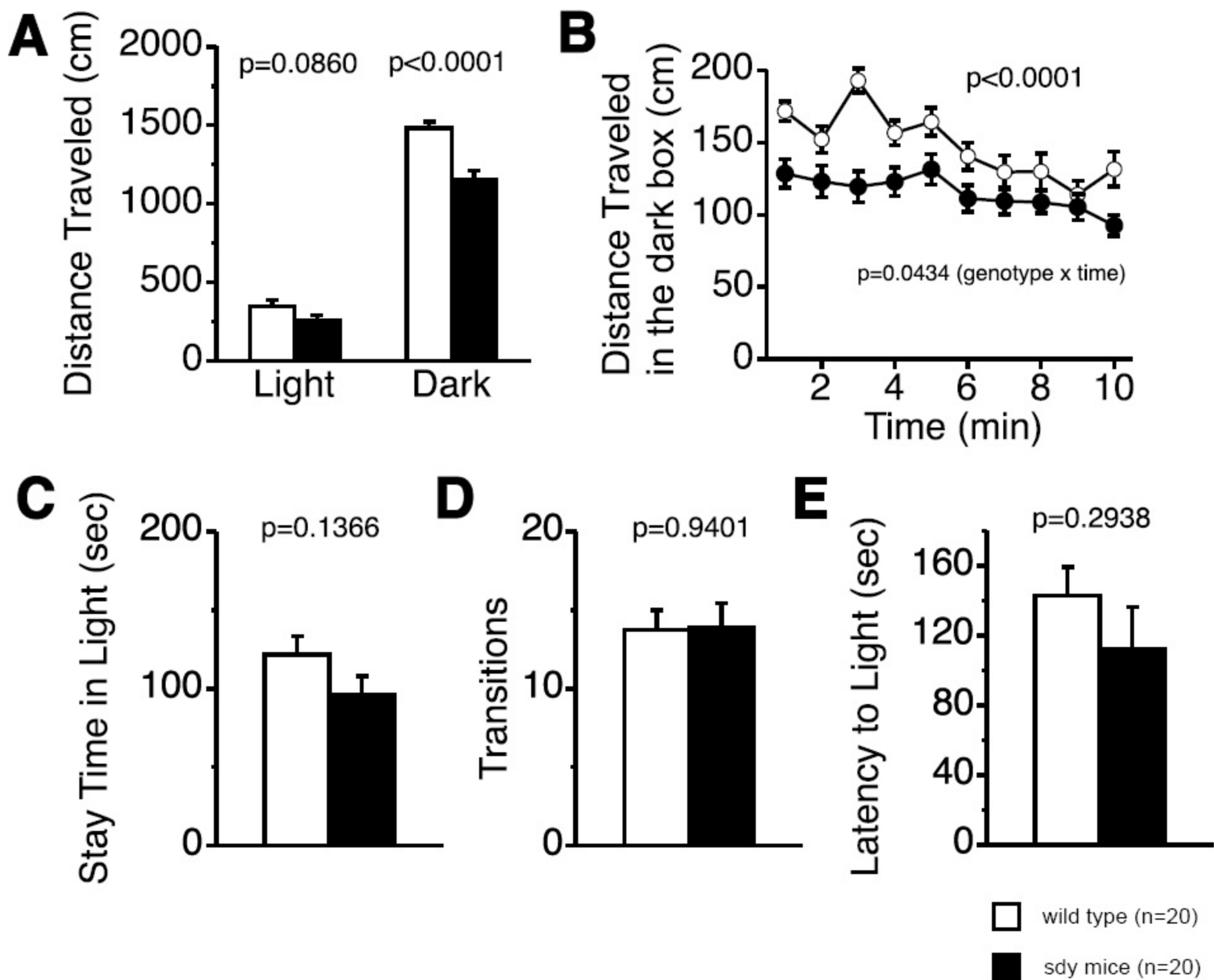

Additional Figure 2

Supplement: Additional file 2 — Reduced locomotor activity in sdy mice in a light/dark transition test. (A) Distance traveled in the light and dark boxes. (B) Time course of the distance traveled in the dark box. (C) Time spent in the light box. (D) Number of transitions between the light and dark boxes. (E) Latency of first entry into light box. Data were analyzed by two-way ANOVA and two-way repeated measures ANOVA. [file 1756-6606-1-11-S2.pdf]

**A**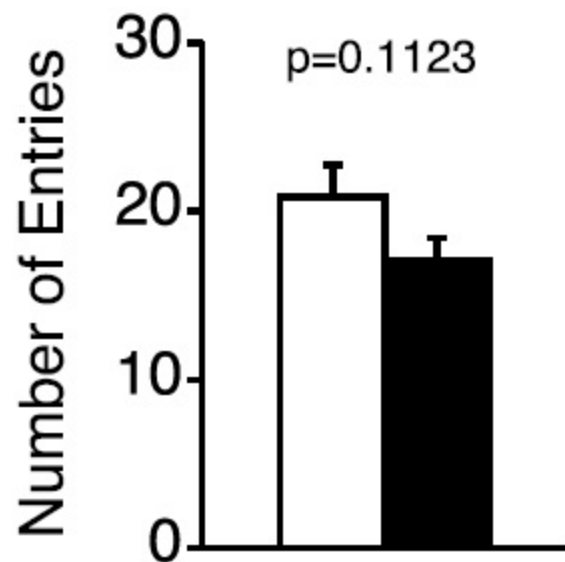**B**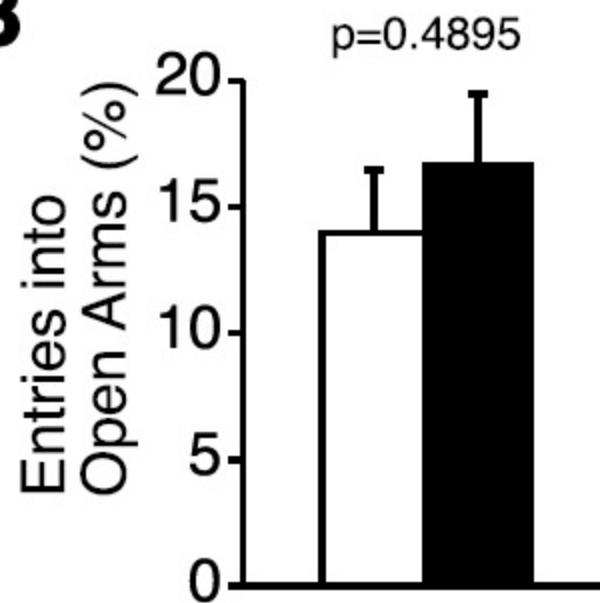**C**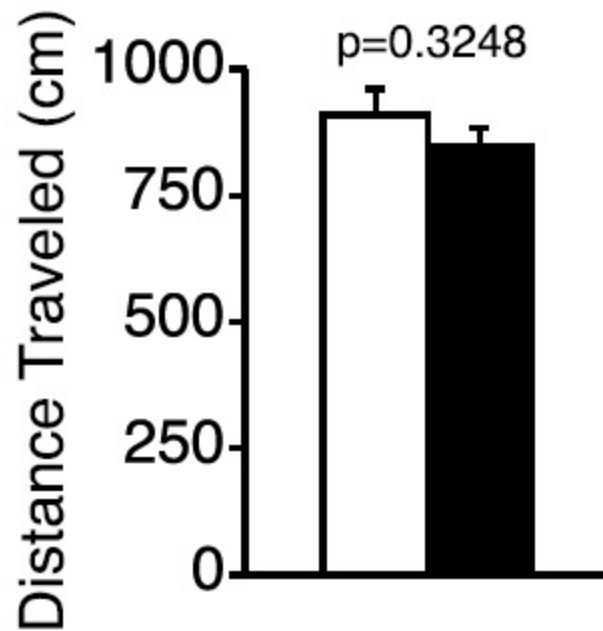**D**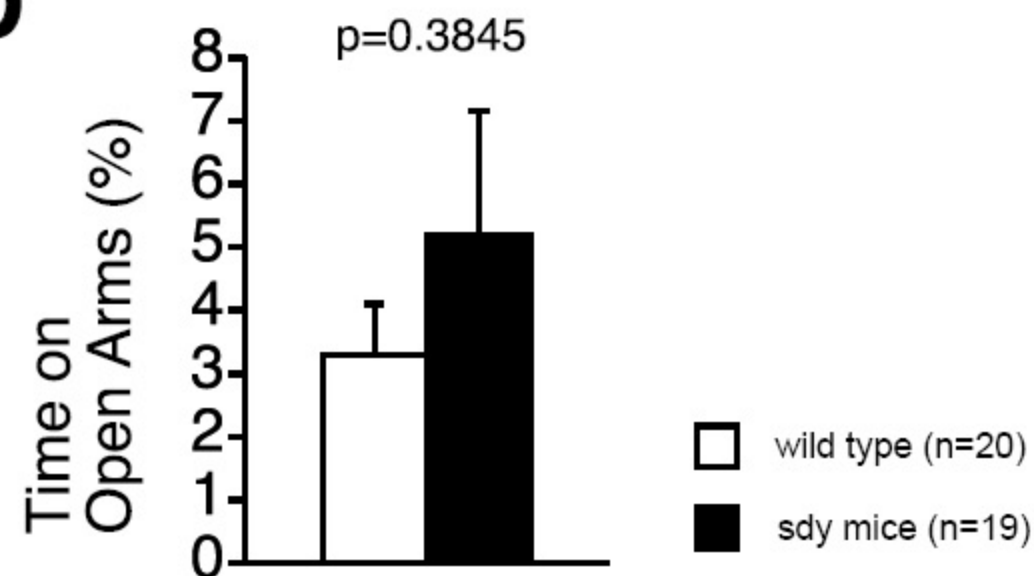

Additional Figure 3

Supplement: Additional file 3 — Normal anxiety-like behavior in sdy mice in elevated plus maze test. (A) Total number of arm entries. (B) Percentage entries into open arms. (C) Distance traveled. (D) Percentage entries into open. Data were analyzed by two-way ANOVA and two-way repeated measures ANOVA. [file 1756-6606-1-11-S3.pdf]
